# Coronary slow flow: role of systemic inflammation and biomarkers in its pathophysiology

**DOI:** 10.1186/s12872-026-05861-2

**Published:** 2026-04-21

**Authors:** Myrna Vianney Muñoz Flores, Arguiñe Ivonne Urraza Robledo, Faviel Francisco González Galarza, Fany Karina Segura López, Héctor Alberto Delgado Aguirre, Alberto Esteban Bazzoni Ruiz, Javier Navarro Pérez Macedo, Aristóteles Ramírez Salazar, Francisco Carlos López Márquez

**Affiliations:** 1 Instituto Mexicano del Seguro Social, Unidad Médica de Alta Especialidad N° 71, Torreón Coahuila, México; 2 Departamento de Inmunobiología Molecular, Centro de Investigación Biomédica, Universidad Autónoma de Coahuila, Torreón Coahuila, México; 3 Escuela de Ingeniería y Ciencias, Tecnológico de Monterrey, Torreón, Coahuila, México; 4 Instituto de Seguridad y Servicios Sociales de los Trabajadores del Estado, Torreón, Coahuila, Mexico

**Keywords:** Coronary slow flow, Systemic inflammation, Coronary angiography, Inflammatory indices

## Abstract

**Background:**

Coronary slow flow (CSF) is characterized by reduced coronary blood flow velocity in the absence of significant obstruction, and it has been associated with systemic inflammation and endothelial dysfunction. This study aimed to evaluate the association between inflammatory indices and the presence of CSF in patients undergoing coronary angiography.

**Methods:**

An analytical cross-sectional study with an exploratory design was conducted in 77 patients undergoing coronary angiography, including those with CSF (*n* = 43) and controls with angiographically normal coronary arteries (*n* = 34). Clinical, biochemical, and hematological variables, TNF-α levels, and inflammatory indices (TyG, NLR, LMR, NPR, PIV, and SIRI) were assessed. Appropriate statistical tests and an exploratory multivariate logistic regression model were performed to evaluate associations with CSF.

**Results:**

The prevalence of CSF was 2.6%, with hypertension and dyslipidemia being the most frequent comorbidities. Patients with CSF exhibited significantly higher levels of total cholesterol (*p* = 0.024), LDL cholesterol (*p* = 0.016), monocytes (*p* = 0.017), and neutrophils (*p* = 0.015). Inflammatory indices TyG, NLR, NPR, PIV, and SIRI were significantly elevated in the CSF group (*p* < 0.05), whereas LMR was lower. No significant differences were found in TNF-α levels, although elevated values were observed in both groups. Multivariate logistic regression analysis did not identify statistically significant independent predictors of coronary slow flow.

**Conclusions:**

CSF appears to represent a systemic inflammatory and microvascular phenotype rather than an isolated angiographic finding. Patients with CSF exhibited higher values of integrated inflammatory indices, suggesting a coordinated cardiometabolic inflammatory profile. These indices may serve as practical and accessible complementary markers associated with CSF; however, they should be considered hypothesis-generating rather than validated diagnostic tools. Larger prospective multicenter studies are required to confirm these associations and their clinical implications.

**Supplementary Information:**

The online version contains supplementary material available at 10.1186/s12872-026-05861-2.

## Background

Cardiovascular diseases (CVDs) represent a major global public health problem. According to the World Health Organization (WHO), they are the leading cause of death worldwide, accounting for 17.9 million annual deaths [1]. Among CVDs, coronary slow flow (CSF) is characterized by reduced coronary blood flow velocity in the absence of significant obstructive coronary artery disease [2]. Although the underlying pathophysiological mechanisms of CSF remain unclear [3], it has been observed in patients with anginal symptoms [4], underscoring its clinical relevance and impact on patient quality of life.

Inflammatory processes are central to the pathophysiology of CSF, as they contribute to endothelial dysfunction, microvascular injury, and altered coronary flow regulation [5]. Cytokines such as interleukins, interferons, and tumor necrosis factor-alpha (TNF-α) have been implicated in vascular inflammation and endothelial activation; however, their diagnostic specificity for CSF remains limited and variable among studies [6–9].

Consequently, recent studies have emphasized the role of hematological inflammatory indices derived from routine laboratory parameters, which may provide a more integrated and stable reflection of systemic inflammation. Examples include the neutrophil-to-lymphocyte ratio (NLR) [10], lymphocyte-to-monocyte ratio (LMR) [11], neutrophil-to-platelet ratio (NPR) [12], pan-immune-inflammation value (PIV) [13], and systemic inflammatory response index (SIRI) [14]. These indices are closely associated with inflammation and cardiovascular risk. Elevated NLR levels have been reported in patients with coronary artery disease and CSF, linking them to atherosclerosis, endothelial dysfunction, and inflammation [10]. Similarly, SIRI, together with NLR, has been associated with cardiovascular mortality in postmenopausal women with osteoporosis and osteopenia [14]. On the other hand, LMR has been correlated with the severity of coronary atherosclerosis [11], while NPR has been shown to increase in patients with ischemic stroke [12]. Additionally, the triglyceride–glucose index (TyG index) is used for the early identification of individuals at high risk of cardiovascular events [15].

The elevation of inflammatory markers in patients with CSF may reflect endothelial activation and inflammation, processes that are integral to the pathological pathways involved in this condition [16]. Therefore, this study aimed to evaluate the association between inflammatory indices and the presence of CSF in patients undergoing coronary angiography. We hypothesized that patients with CSF would exhibit higher levels of inflammatory indices compared with those with normal coronary flow.

## Methods

### Study population

**Fig. 1:**
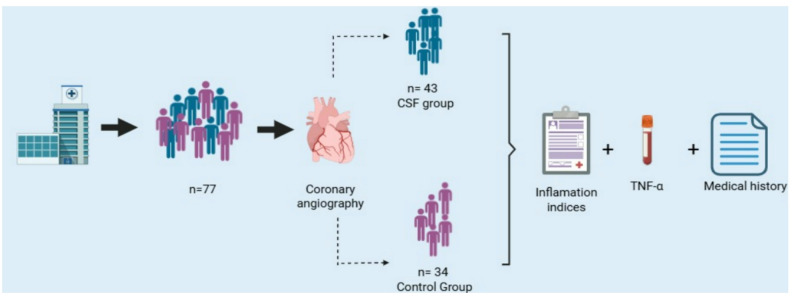
Flowchart of patient selection and study design

This study was conducted at the Mexican Social Security Institute (Instituto Mexicano del Seguro Social, IMSS), in the High Specialty Medical Unit Hospital de Especialidades No. 71 (UMAE HE No. 71), located in Torreón, Coahuila, from June 2023 to June 2024. The study included patients who underwent coronary angiography in the hemodynamics unit and were diagnosed with CSF (case group), as well as those with angiographically normal coronary arteries (control group). To test the proposed hypothesis, the formula for calculating the sample size based on the difference in proportions was applied. The analysis was conducted with a significance level of 95% (α = 0.05) and a statistical power of 80% (β = 0.20), resulting in an estimated sample size of 36 subjects per group. (Fig. 1) Eligible participants were adults over 18 years of age, of either sex, undergoing coronary angiography for the first time. Patients were excluded if they had a history of diagnosed cardiovascular disease, known coronary lesions, prior acute myocardial infarction, previously documented acute coronary syndrome, autoimmune or chronic inflammatory disorders, cancer, trauma, or major surgery within the past 6 months, to minimize potential bias in inflammatory measurements. Patients receiving systemic corticosteroids or other immunomodulatory therapy were not systematically recorded and this has been acknowledged as a limitation. Patients were included consecutively according to the availability of complete clinical and laboratory data during the study period. Controls were selected consecutively from patients with angiographically normal coronary arteries during the same period, provided they met the inclusion criteria. An analytical cross-sectional study with an exploratory design was conducted. The distribution of cases and controls reflects the real frequency of CSF among patients undergoing coronary angiography at our institution.

The study was approved by the Research Ethics Committee and the Local Health Research Committee No. 501 at UMAE HE No. 71 (Approval No.: R-2023-501-046). All participants received detailed information about the study, and written informed consent was obtained. The research was conducted in accordance with the principles of the Declaration of Helsinki and the Belmont Report.

### Coronary angiography

Coronary angiography was performed via femoral or radial arterial access using the Seldinger technique, with superficial and deep local anesthesia (2% lidocaine) prior to arterial puncture. Iopamidol, a low-osmolar contrast medium, was used during the procedure. The diagnosis of CSF was based on the definition proposed by Beltrame [17], which requires the absence of significant angiographic lesions (≥ 40%) and delayed distal vessel contrast opacification corresponding to TIMI grade 2 flow (i.e., requiring three cardiac cycles to fully opacify the vessel). In contrast, patients with TIMI grade 3 flow and no obstruction or alterations in major epicardial arteries were classified as having “angiographically normal arteries.” All angiographic results were interpreted and validated by two interventional cardiologists.

### Biochemical and hematological parameters

Biochemical and hematological parameters were obtained from patient medical records. Prior to coronary angiography, patients underwent laboratory testing, including blood chemistry, lipid profile, and complete blood count. Biochemical analyses were performed using the Vitros^®^ 4600 automated dry chemistry analyzer (Ortho Clinical Diagnostics, Raritan, NJ, USA), and hematological parameters were measured with the XN-1000^®^ automated analyzer (Sysmex Corporation, Kobe, Japan), following the manufacturers’ specifications.

### Determination of TNF-α

Quantification of TNF-α was performed using a quantitative sandwich enzyme-linked immunosorbent assay (ELISA) for human TNF-α (lot: 20114909, code: SK00109-01, Aviscera Bioscience). Reagents, including wash solution, TNF-α standard solution, positive control, detection antibody concentrate, and streptavidin-HRP conjugate, were prepared according to the manufacturer’s instructions. Microplate readings were conducted with the Stat Fax 4700 reader at 450 nm. According to the manufacturer’s specifications [18], the detection range was 1.96–500 pg/mL, with an expected normal serum concentration below 15 pg/mL. All samples were collected in fasting conditions, prior to coronary angiography.

### Statistical analysis

Descriptive statistical analysis was performed. Qualitative variables were expressed as absolute frequencies and percentages. Normality of quantitative variables was assessed using the Shapiro–Wilk test. Quantitative variables were reported as mean ± standard deviation or as median and interquartile range, depending on distribution. Comparisons between the two study groups were performed using the chi-square test for qualitative variables, and either Student’s t-test or the Mann–Whitney U test for quantitative variables, according to distribution.

Variables showing a *p*-value < 0.05 in univariate analysis and/or those considered clinically relevant based on previous literature were included in the multivariate logistic regression model. A backward stepwise selection method was applied to explore independent associations with CSF, and odds ratios (OR) with 95% confidence intervals (CI) were calculated. Statistical analysis was performed using SPSS version 26 (IBM Corp., Armonk, NY, USA). A *p*-value < 0.05 was considered statistically significant. No post hoc power analysis was performed.

## Results

The present study was conducted between July 2023 and July 2024. During this period, a total of 1,650 coronary angiographies were performed, of which 2.6% (*n* = 43) of patients were diagnosed with CSF. After applying inclusion and exclusion criteria, the final sample consisted of 77 participants (43 with CSF and 34 without CSF); 53% were male (Table 1). The mean age of participants was 62 ± 9.57 years. The mean body mass index (BMI) was 29.13 ± 4.80 kg/m², while the mean heart rate was 70.83 ± 12.51 beats per minute. Hypertension was the most prevalent comorbidity (82%), followed by dyslipidemia (41%) and diabetes mellitus (37%). Additionally, 45% of patients were smokers. No statistically significant differences were observed between the CSF and control groups in sociodemographic, clinical, or anthropometric characteristics (Table 1).

**Table 1:** Sociodemographic, clinical, and anthropometric characteristics

Parameters	*N* = 77	CSF Group (*n* = 43)	Control Group (*n* = 34)	*p*-value
Sex				0.428
Male *n*(%)	41(53)	22(51)	19(56)	
Female *n*(%)	36(47)	21(49)	15(44)	
Age (years)	62 ± 9.57	63 ± 9	62 ± 9	0.612
BMI (kg/m^2^)	29.13 ± 4.80	28.80 ± 4.64	29.55 ± 5.05	0.286
Heart rate (bpm)	70.83 ± 12.51	70.32 ± 12.19	71.47 ± 13.05	0.821
Hypertension *n*(%)	64(82)	38(88)	26(76)	0.141
Dyslipidemia *n*(%)	32(41)	20(46)	11(32)	0.224
Diabetes *n*(%)	29(37)	18(42)	11(32)	0.269
Smoking *n*(%)	35(45)	19(44)	16(47)	0.491

The results are expressed as mean±standard deviation

*BMI* body mass index, *bpm *beats per minute, *CSF * coronary slow flow

Biochemical and hematological analyses revealed significant differences between groups. Total cholesterol levels were significantly higher in the CSF group (165 [153.00–186.00] vs. 139 [118.00–178.50]; *p* = 0.024), as were LDL cholesterol levels (96.16 ± 29.18 vs. 80.29 ± 26.82; *p* = 0.016). Monocyte counts were also higher in the CSF group (0.64 ± 0.12 vs. 0.57 ± 0.15; *p* = 0.017), as were neutrophil counts (4.40 [4.00–5.30] vs. 4.00 [3.53–4.70]; *p* = 0.015) (Table 2).

**Table 2:** Biochemical and hematological parameters

Parameter	CSF Group (*n* = 43)	Control Group (*n* = 34)	*p*-value
Glucose (mg/dL)	100.00 (91–121)	90.50 (81.50–114.00)	0.073
BUN (mg/dL)	16.86 (12.60-20.35)	14.00 (12.00-18.19)	0.353
Creatinine (mg/dL)	0.90 (0.70-1.00)	0.80 (0.60-1.00)	0.283
Uric acid (mg/dL)	5.17 ± 1.50	5.29 ± 1.82	0.748
Triglycerides (mg/dL)	147.00 (110.00-200.00)	115.50 (93.00-167.50)	0.054
Cholesterol (mg/dL)	**165.00(153.00-186.00)**	**139.00 (118.00-178.50)**	0.024*
HDL (mg/dL)	39.99 ± 10.78	39.69 ± 10.50	0.901
LDL (mg/dL)	**96.16 ± 29.18**	**80.29 ± 26.82**	0.016*
VLDL (mg/dL)	29.40 (22.00–40.00)	23.10 (18.60–33.50)	0.054
Leucocytes (10³/µL)	7.20 (6.70–8.02)	7.19 (6.19–8.60)	0.898
Lymphocytes (10³/µL)	1.78 (1.58–2.20)	1.99 (1.60–2.81)	0.133
Monocytes (10³/µL)	**0.64 ± 0.12**	**0.57 ± 0.15**	0.017*
Eosinophils (10³/µL)	0.11 (0.10–0.20)	0.12 (0.10–0.20)	0.688
Basophils (10³/µL)	0.04 (0.03–0.06)	0.04 (0.02–0.05)	0.331
Neutrophils (10³/µL)	**4.40 (4.00-5.30)**	**4.0 (3.57–4.70)**	0.015*
Erythrocytes (10³/µL)	4.64 ± 0.64	4.85 ± 0.55	0.129
Hemoglobin (g/dL)	13.86 ± 1.96	14.34 ± 1.77	0.277
Hematocrit (%)	41.68 ± 5.79	43.40 ± 5.45	0.188
MCV (fL)	89.87 ± 5.62	89.43 ± 6.46	0.752
MCH (pg)	30.11 ± 2.28	29.59 ± 2.66	0.361
MCHC (g/dL)	33.10 ± 1.58	33.05 ± 1.28	0.881
Platelets (10³/µL)	231.72 ± 66.58	248.44 ± 64.60	0.271

The results are expressed as mean±standard deviation or median (interquartile range) according to their distribution

*BUN *urea nitrogen, *HDL *high-density lipoprotein, *LDL *low-density lipoprotein, *VLDL *very-low-density lipoprotein, *MCV *mean corpuscular volume, *MCH *mean corpuscular hemoglobin, *MCHC *mean corpuscular hemoglobin concentration, *CSF * coronary slow flow

* *p*-value < 0.05 indicates a significant difference between groups

No statistically significant differences were found in TNF-α levels; however, values were elevated above the normal range in both groups (Table 3). Analysis of indices associated with inflammation and insulin resistance, including TyG, NLR, LMR, NPR, PIV, and SIRI, showed significant differences, with higher values in the CSF group, suggesting a heightened inflammatory response and greater insulin resistance (Table 3).

**Table 3:** Inflammation parameters

Parameters	CSF Group (*n* = 43)		Control Group (*n* = 34)	*p*-value
TNF-α (pg/ml)	46.21 (16.91–60.02)		54.13 (14.15–62.51)	0.704
TyG Index	9.03 ± 0.52		8.76 ± 0.61	0.042*
NLR	2.77 ± 1.15		2.06 ± 0.70	0.002*
LMR	2.99 ± 0.98		4.13 ± 1.85	0.001*
NPR	19.15(15.85–27.83)		16.60(11.86–20.94)	0.009*
PIV	353.16(273.69–516.60)		265.76(174.17-377.24)	0.006*
SIRI	1.49(1.20–2.28)		1.17(0.73–1.39)	< 0.001*

The results are expressed as mean±standard deviation or median (interquartile range) according to their distribution

*TNF-α *tumor necrosis factor-alpha, *TyG Index *riglyceride/glucose index, *NLR *neutrophil-to-lymphocyte ratio, *LMR *lymphocyte-to-monocyte ratio, *NPR *neutrophil-to-platelet ratio, *PIV *pan-immune-inflammation value, *SIRI *systemic inflammatory response index, *CSF * coronary slow flow

* *p*-value < 0.05 indicates a significant difference between groups

Multivariate logistic regression analysis including inflammatory indices and lipid parameters did not reveal statistically significant independent predictors of coronary slow flow *p* > 0.05 (Table 4). This finding is likely influenced by the modest sample size and the intercorrelation among inflammatory indices. The Hosmer–Lemeshow goodness-of-fit test demonstrated adequate model calibration (χ² = 6.796, *p* = 0.559).

**Table 4:** Logistic regression analysis between inflammatory indices with slow coronary flow

	OR	95%CI	*p*-value
SIRI	0.670	0.012–36.767	0.845
Cholesterol	1.022	0.973–1.073	0.390
LDL	0.999	0.948–1.053	0.970
NLR	1.534	0.431–5.453	0.509
LMR	0.492	0.184–1.319	0.159
NPR	1.043	0.936–1.163	0.443
PIV	1.001	0.992–1.009	0.890
TyG Index	2.362	0.676–8.250	0.178

*SIRI* systemic inflammatory response index, *LDL *low-density lipoprotein, *NLR *neutrophil-to-lymphocyte ratio, *LMR *lymphocyte-to-monocyte ratio, *NPR *neutrophil-to-platelet ratio, *PIV *pan-immune-inflammation value, *TyG Index *triglyceride/glucose index

## Discussion

Coronary slow flow (CSF) is a cardiac phenomenon that has been linked to biochemical and hematological alterations. However, its pathophysiology remains unclear [19]. The prevalence of CSF observed in this study was 2.6%, which falls within the range reported in the literature (1–7% of patients undergoing coronary angiography) [20]. Nonetheless, some studies have reported higher rates, such as 23.7% in an Indian population [21], underscoring the variability of this condition across different populations and geographic contexts.

CSF has been associated with inflammatory processes that impair endothelial function [16]. Obesity may contribute to this mechanism through chronic low-grade metabolic inflammation and endothelial dysfunction [22–24]. In our study, both groups presented BMI values consistent with overweight, suggesting a shared proinflammatory background; however, inflammatory indices were significantly higher in the CSF group, reinforcing the potential role of immune–metabolic imbalance in this phenomenon.

Lipid abnormalities also play a key role in vascular inflammation. Elevated cholesterol and LDL levels impair nitric oxide bioavailability, promote oxidative modification of LDL, and trigger endothelial injury and microcirculatory dysfunction [25–27]. In our cohort, total cholesterol and LDL were significantly higher in CSF patients, in line with previous studies [28].

Monocytes and neutrophils, central mediators of innate immunity, are directly involved in atherogenesis and vascular inflammation [29–32]. Their increased counts in CSF patients, consistent with previous findings [33], likely reflect both systemic inflammation and direct endothelial injury, supporting their potential value as prognostic biomarkers in CSF.

Various inflammatory biomarkers have been associated with cardiovascular diseases, among them tumor necrosis factor-alpha (TNF-α). This cytokine is recognized as a biological mediator of metabolic dysfunction and vascular inflammation [34–36]. However, in the present study, TNF-α levels did not differ significantly between the CSF and non-CSF groups, although concentrations were elevated in both. This pattern likely reflects a background of chronic metabolic inflammation rather than a CSF-specific process. Previous studies have reported increased TNF-α levels in CSF after percutaneous coronary intervention for non–ST-segment elevation acute coronary syndrome [37], suggesting its role in vascular inflammation. The persistently high concentrations observed in both groups may reflect chronic systemic inflammation that contributes to endothelial dysfunction [38]. Possible confounding factors —such as metabolic syndrome, obesity, insulin resistance, or subclinical coronary atherosclerosis— may explain these elevated values across groups. Indeed, TNF-α is closely linked to adipose tissue–derived inflammation and endothelial dysfunction, mechanisms that can occur independently of angiographically visible disease [22, 36]. Consequently, while TNF-α remains a relevant proinflammatory cytokine, its diagnostic or discriminatory utility for CSF appears limited when compared to composite inflammatory indices such as SIRI, PIV, or NLR, which integrate multiple immune cell components and better capture systemic inflammatory activity. Future studies should examine whether TNF-α participates indirectly by amplifying pathways represented in these broader indices rather than serving as an isolated biomarker of CSF.

Furthermore, extracoronary vascular alterations have been described in patients with CSF, including decreased carotid blood flow velocities and disturbed hemodynamic patterns, supporting the presence of systemic endothelial dysfunction beyond the coronary circulation [39].

In this context, composite inflammatory indices provide a more integrated and clinically meaningful assessment of systemic inflammation in patients with suspected coronary microvascular dysfunction. The TyG index, NLR, LMR, NPR, PIV, and SIRI are important markers associated with inflammation and cardiovascular risk, with established roles in cardiovascular disease. NLR has been shown to be elevated in patients with CSF, coronary artery disease, and coronary artery ectasia compared with angiographically normal individuals [19]. Conversely, LMR has been inversely correlated with the prevalence and severity of cardiovascular diseases such as aortic dilatation [40]. NPR has been identified as a predictor of short-term mortality in patients with ST-segment elevation myocardial infarction [41]. Elevated PIV and SIRI have demonstrated associations and predictive value in CSF patients [42, 43]. In this study, significant differences were observed between groups in TyG (CSF: 9.03 ± 0.52 vs. control: 8.76 ± 0.61; *p* = 0.042), NLR (CSF: 2.77 ± 1.15 vs. control: 2.06 ± 0.70; *p* = 0.002), NPR (CSF: 19.15 [15.85–27.83] vs. control: 16.60 [11.86–20.94]; *p* = 0.009), PIV (CSF: 353.16 [273.69–516.60] vs. control: 265.76 [174.17–377.24]; *p* = 0.006), and SIRI (CSF: 1.49 [1.20–2.28] vs. control: 1.17 [0.73–1.39]; *p* < 0.001), with higher values in the CSF group. In contrast, LMR levels were higher in the control group compared with CSF patients (CSF: 2.99 ± 0.98 vs. control: 4.13 ± 1.85; *p* < 0.001), demonstrating an inverse relationship between groups. This may be explained by the role of neutrophils as first responders in inflammation, infiltrating endothelial tissue and releasing pro-oxidant and proinflammatory mediators [44]. Patients with CSF exhibited increased neutrophil counts and decreased lymphocyte counts. These indices may provide a comprehensive picture of the inflammatory state and could serve as useful tools for cardiovascular risk assessment.

Previous studies have also linked biochemical and metabolic markers to coronary slow flow, such as the uric acid to HDL-C ratio, which reflects oxidative stress and endothelial dysfunction [45]. Likewise, improvement of microvascular perfusion with trimetazidine therapy supports the role of metabolic modulation in CSF [46].

Our findings align with emerging evidence that coronary slow flow (CSF) is not merely an angiographic anomaly but a manifestation of systemic inflammation and microvascular dysfunction. Recent studies have demonstrated that composite inflammatory indices (SIRI and PIV) are associated with CSF and can differentiate affected patients from those with normal coronary arteries, even without epicardial stenosis. These indices integrate neutrophil-, monocyte-, lymphocyte-, and platelet-mediated activity, thereby providing a more comprehensive assessment of low-grade immune activation, endothelial injury, and microvascular impairment than isolated cytokine measurements [42, 43, 47].

In our cohort—representing one of the first Latin American populations in which integrated inflammatory indices have been systematically evaluated in relation to CSF—higher values of TyG, NLR, NPR, PIV, and SIRI, together with dyslipidemia, were consistently observed in patients with CSF, whereas TNF-α levels did not discriminate between groups.

These findings highlight the novelty and clinical relevance of our study and support the concept that CSF reflects a chronic proinflammatory and cardiometabolic state contributing to diffuse endothelial dysfunction rather than a focal flow disturbance.

From a clinical perspective, integrated inflammatory indices derived from routine hematologic and biochemical parameters may provide complementary information in patients presenting with angina and angiographically normal coronary arteries. Because these indices are easily calculated from standard laboratory tests, they represent low-cost, accessible, and reproducible tools that may help characterize the inflammatory and metabolic profile associated with coronary slow flow or microvascular dysfunction. However, given the relatively small sample size of this study, these inflammatory indices should be considered hypothesis-generating tools. Given the analytical cross-sectional and exploratory design, these findings should be interpreted as non-causal, reflecting observed differences rather than independent associations. Future studies should determine their prognostic utility and evaluate whether index-based strategies improve risk stratification and clinical outcomes in patients with CSF.

## Limitations

This study provides information on the behavior of inflammatory indices in patients with coronary slow flow (CSF). Although the logistic regression model followed the “events per variable” (EPV) rule to minimize overfitting, the relatively small sample size limits statistical power, generalizability, and increases the risk of bias. Consequently, the multivariate findings should be interpreted with caution. Larger sample sizes and prospective multicenter studies are required to confirm these associations and to allow a reliable evaluation of the predictive and prognostic value of inflammatory indices in CSF.

Although control participants were classified as having angiographically normal coronary arteries, microcirculatory dysfunction cannot be excluded. The absence of specific assessments such as coronary flow reserve or endothelial function testing may have allowed inclusion of individuals with subclinical microvascular alterations, potentially influencing inflammatory comparisons.

CSF diagnosis was based on qualitative TIMI flow grading according to Beltrame (Circ J, 2012); quantitative assessments such as corrected TIMI frame count or coronary flow reserve were not performed due to institutional limitations. In addition, vessel-specific angiographic characterization and intracoronary imaging were not systematically available, precluding formal assessment of coronary spasm or subclinical plaque morphology. Future studies should incorporate quantitative microvascular evaluation to enhance diagnostic accuracy and pathophysiological interpretation.

## Conclusion

This study suggests that coronary slow flow (CSF) may reflect a systemic inflammatory and microvascular phenotype rather than an isolated angiographic finding. Both groups exhibited evidence of low-grade inflammation, but patients with CSF showed higher SIRI, TyG, NLR, NPR, and PIV values, indicating a potential cardiometabolic profile characterized by coordinated immune activation and endothelial dysfunction.

These findings highlight the potential relevance of integrated inflammatory indices as practical and accessible complementary biomarkers in patients with CSF. However, given the analytical cross-sectional and exploratory design, these markers should be considered hypothesis-generating rather than validated diagnostic instruments.

Future multicenter prospective studies are warranted to confirm these findings and to explore their clinical implications. 

## Supplementary Information

Supplementary Material 1: Table S1. Logistic regression analysis between biochemical, hematological variables, and systemic inflammatory response index with coronary slow flow

Supplementary Material 1

## Data Availability

The datasets used and/or analyzed during the present study are available from the corresponding author on reasonable request.

## Abbreviations

BMI: Body mass index
BUN: Urea nitrogen
CSF: Coronary slow flow
CVDs: Cardiovascular diseases
ELISA: Enzyme-linked immunosorbent assay
HDL: High-density lipoprotein
LDL: Low-density lipoprotein
LMR: Lymphocyte-to-monocyte ratio
MCH: Mean corpuscular hemoglobin
MCHC: Mean corpuscular hemoglobin concentration
MCV: Mean corpuscular volume
NLR: Neutrophil-to-lymphocyte ratio
NPR: Neutrophil-to-platelet ratio
OR: Odds ratios
PIV: Pan-immune-inflammation value
SIRI: Systemic inflammatory response index
TNF-α: Tumor necrosis factor-alpha
TyG: Triglyceride–glucose index
VLDL: Very-low-density lipoprotein
