# Supplementary material for: Coronary slow flow: role of systemic inflammation and biomarkers in its pathophysiology
Source: BMC Cardiovasc Disord. 2026 Apr 21;26:472. doi: 10.1186/s12872-026-05861-2 (PMC13231589; doi:10.1186/s12872-026-05861-2)
Supplement: Supplementary file 1 — Supplementary Material 1: Table S1. Logistic regression analysis between biochemical, hematological variables, and systemic inflammatory response index with coronary slow flow [file 12872_2026_5861_MOESM1_ESM.docx]

**Supplementary Material**

| **Table S1. Logistic regression analysis between biochemical, hematological variables, and systemic inflammatory response index with slow coronary flow** | | | |
| --- | --- | --- | --- |
|  | **OR** | **95%CI** | **p-value** |
| **SIRI** | **4.985** | **1.761 - 14.112** | **0.002** |
| **Cholesterol** | **1.039** | **1.002 -1.077** | **0.040** |
| LDL | 0.989 | 0.941-1.018 | 0.979 |
| LDL: low-density lipoprotein; SIRI: systemic inflammatory response index. | | | |
