# Supplementary figures and images for: Coronary slow flow: role of systemic inflammation and biomarkers in its pathophysiology
Source: BMC Cardiovasc Disord. 2026 Apr 21;26:472. doi: 10.1186/s12872-026-05861-2 (PMC13231589; doi:10.1186/s12872-026-05861-2)

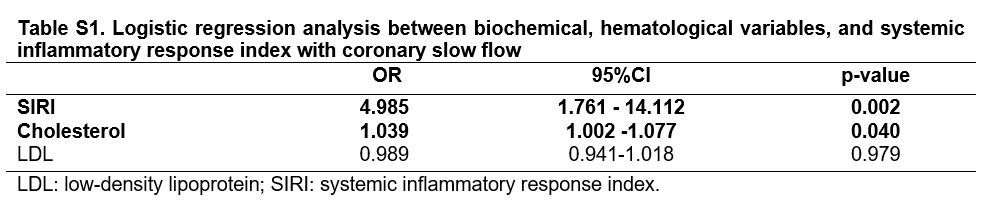

Supplement: Supplementary file 2 — Supplementary Material 1 [file 12872_2026_5861_MOESM2_ESM.jpeg]
